# Impact of allergic bronchopulmonary aspergillosis overlap in chronic pulmonary aspergillosis

**DOI:** 10.1183/23120541.01540-2025

**Published:** 2026-09-21

**Authors:** Ana Raquel Afonso, Dareen Marghlani, Mohammed Alramadhan, Aran Singanayagam, Melody Ni, Darius Armstrong-James, Anand Shah

**Affiliations:** 1 Department of Pulmonology, Unidade Local de Saúde de Trás-os-Montes e Alto Douro, Hospital de Vila Real, Vila Real, Portugal; 2 Centre of Bacterial Resistance Biology, Department of Infectious Disease, Imperial College London, London, UK; 3 Centre for Bacterial Resistance Biology, Royal Brompton and Harefield Hospitals, Guy's and St Thomas’ NHS Foundation Trust, London, UK; 4 NIHR HealthTech Research Centre in In Vitro Diagnostics, Department of Surgery and Cancer, Faculty of Medicine, Imperial College London, London, UK; 5 Imperial Fungal Science Network, Department of Infectious Disease, Imperial College London, London, UK

## Abstract

**Background:**

Chronic pulmonary aspergillosis (CPA) is a destructive fungal infection caused by *Aspergillus fumigatus*, leading to significant morbidity in individuals with structural lung disease. Clinical and immunological overlap with allergic bronchopulmonary aspergillosis (ABPA) has been recognised, but its extent and prognostic relevance remain uncertain. This study assessed ABPA features in CPA and their relationship with immunological markers and long-term outcome.

**Methods:**

We conducted a retrospective cohort study including individuals with confirmed CPA at the Royal Brompton Hospital until December 2023. Diagnoses followed ERS/ESCMID and 2024 ISHAM criteria. Demographic, clinical, microbiological and immunological data were analysed, and group comparisons performed using logistic regression and Cox proportional hazards models.

**Results:**

Among 166 individuals with CPA, 45 (27%) met ABPA diagnostic criteria. CPA/ABPA overlap was independently associated with asthma (OR 10.16) and pan-azole resistance (OR 19.37), and inversely with sarcoidosis (OR 0.22). Overall 5-year survival was 82% (84.6% in CPA alone *versus* 76.6% in overlap; p=0.44). Older age, lower body mass index and albumin, and elevated *A. fumigatus*-specific IgE and IgG were associated with higher mortality, while longitudinal increase in *A. fumigatus*-specific IgE was also linked to worse outcome. Low serum albumin independently predicted mortality (HR 0.72; p=0.001).

**Discussion:**

CPA/ABPA overlap represents a distinct clinical phenotype linked to airway disease, antifungal resistance and Th2-driven inflammation. Nutritional status and immunological activity, particularly rising *A. fumigatus*-specific IgE, emerged as key prognostic markers, linking type 2 inflammation to disease progression. These findings highlight the need for phenotype-based risk stratification and exploration of targeted immunomodulatory strategies in CPA.

## Shareable abstract

**CPA/ABPA overlap represents a distinct clinical phenotype in individuals with CPA associated with underlying airway disease, antifungal resistance and Th2-driven inflammation, with rising biomarkers linked to disease progression** https://bit.ly/4eR5Xwv

## Introduction

Chronic pulmonary aspergillosis (CPA) is a progressive fungal infection of the lung caused mainly by *Aspergillus fumigatus* affecting individuals with pre-existing lung conditions such as prior tuberculosis, COPD, nontuberculous mycobacterial pulmonary disease (NTM-PD), bronchiectasis or sarcoidosis [1]. CPA is associated with substantial morbidity and poor prognosis, representing a major global health burden, with an estimated 1.8 million new cases and over 300 000 deaths annually [2, 3]. A recent systematic review estimated a pooled mortality rate of 27%, with 1- and 5-year mortality of 15% and 32%, respectively, although with significant study heterogeneity [3]. Prognosis variability appears to be influenced by host immune status, CPA subtype, pulmonary involvement, healthcare access and timing of antifungal therapy, with clinical course ranging from indolent to rapidly progressive disease [3]. Specific predictors of mortality, however, remain poorly defined, with limited understanding of underlying pathophysiology [3, 4].

Emerging research has suggested a closer relationship between CPA and allergic bronchopulmonary aspergillosis (ABPA), an immunologically mediated disorder driven by hypersensitivity to *Aspergillus* species [5, 6]. Although traditionally considered distinct entities, recent studies indicate coexistence and overlapping immunological features [7, 8]. CPA and ABPA reflect different host immune responses – *Aspergillus* typically inducing a Th2-dominant response in immunocompetent individuals with asthma, leading to ABPA, whereas CPA is thought to arise from defective Th1 responses in structurally abnormal lungs [8]. Some individuals with CPA also display ABPA-associated features such as raised total immunoglobulin E (IgE), *A. fumigatus*-specific IgE and eosinophilia [9]. In one cohort, ∼5% of CPA individuals met full ABPA immunological criteria, and 22% met obligatory criteria [8]. Conversely, elevated *A. fumigatus*-specific IgG, a hallmark of CPA, is also seen in ∼90% of ABPA individuals and forms part of diagnostic guidelines, limiting its discriminatory value [10–13].

Individuals with CPA fulfilling ABPA immunological criteria exhibited higher *A. fumigatus*-specific IgG and more frequent intracavitary mycetoma, suggesting that CPA/ABPA overlap represents a phenotype of heightened immunological activity, higher fungal load and potentially worse outcomes [8]. Even without ABPA, immunological profiling of CPA shows distinct *Aspergillus* sensitisation patterns [14], associated with increased mycetoma and airflow obstruction. Whether CPA/ABPA overlap represents progression along a disease spectrum or a hybrid entity driven by shared risk factors such as immune dysregulation and lung damage remains unclear [1, 14, 15]. Furthermore, little is known about risk factors predisposing to overlap, its effect on outcomes or whether *Aspergillus* sensitisation itself may represent a modifiable trait or therapeutic target [7, 16].

In this study, we performed a single-centre retrospective analysis of a large CPA cohort to determine the prevalence of ABPA overlap and its prognostic impact, focusing on immunological markers, *Aspergillus* sensitisation, and their relationship to long-term outcomes and mortality.

## Methods

We conducted a retrospective cohort study including all individuals with confirmed CPA managed at the Royal Brompton Hospital (RBH) between 2012 and 31 December 2023. Both living and deceased recruits were included if actively followed at RBH. Ethical approval was granted by the UK Health Research Authority (REC ref: 21/HRA/3400). Data were extracted from electronic medical records, including demographic, clinical, radiological, functional, immunological, microbiological and treatment-related variables. Follow-up information comprised antifungal exposure, surgical or interventional radiology procedures and survival outcomes. Individuals were stratified into two groups for analysis: CPA with ABPA features *versus* CPA alone. The study followed STROBE (Strengthening the Reporting of Observational Studies in Epidemiology) guidelines.

CPA diagnosis followed international ERS/ESCMID criteria [10, 17], requiring ≥3 months of: 1) compatible clinical symptoms (*e.g.* cough, haemoptysis, weight loss, malaise, fever or dyspnoea); 2) characteristic thoracic CT findings (cavities with or without fungal ball, nodules, pleural thickening or peri-cavitary infiltrates); 3) evidence of *Aspergillus* infection, direct (positive culture or microscopy from biopsy, sputum or bronchoalveolar lavage fluid (BALF)) or indirect (elevated *A. fumigatus*-specific IgG, positive precipitins or serum/BALF galactomannan above diagnostic thresholds); and 4) exclusion of alternative diagnoses. ABPA was diagnosed per 2024 ISHAM revised criteria [18], requiring a predisposing condition (asthma, cystic fibrosis, COPD or bronchiectasis) or compatible clinicoradiological presentation, plus two essential immunological features: total serum IgE >500 IU·mL^−1^ and *A. fumigatus*-specific IgE ≥0.35 kUA·L^−1^. At least two supportive features were also required, including elevated *A. fumigatus*-specific IgG, eosinophilia >500 cells·μL^−1^ or characteristic imaging (*e.g.* central bronchiectasis, mucus plugging, high-attenuation mucus or transient infiltrates) [18].

Statistical analyses were performed using IBM SPSS Statistics, v24.0. Continuous variables were expressed as mean±standard deviation (sd) or median interquartile range (IQR), and categorical variables as counts and percentages. Group comparisons were conducted using descriptive statistics and binary logistic regression, with results reported as odds ratios (OR) and 95% confidence intervals (CI). A two-sided p-value <0.05 was considered significant. Variables with p<0.10 in univariate analysis were included in multivariate models; those with high variability or limited discriminatory value were excluded. Highly correlated variables were excluded to reduce collinearity.

All selected variables were entered simultaneously into final multivariable models. Missing data were assessed, and cases with incomplete clinical or serological information excluded from corresponding analyses; no imputation performed.

For survival analyses, individuals lost to follow-up were censored at last contact. Serological markers (total IgE, *A. fumigatus*-specific IgE and IgG) were analysed as continuous variables at multiple timepoints (diagnosis, study baseline and final end-point), with temporal variance calculated and included in survival models. A 5-year observation window was defined for each individual. The end-point was death, last contact or 31 December 2023. Deaths within the window were coded as events (status=1), others as censored (status=0). Time-to-event was measured from start of follow-up to end-point or death. Survival probabilities were estimated using Kaplan–Meier curves and compared by log-rank test. Predictors of mortality were assessed using univariate and multivariable Cox regression, with hazard ratios (HRs) and 95% CIs reported. The proportional hazards assumption was verified for all models.

## Results

### General cohort characteristics

A total of 166 individuals with CPA were included, of whom 45 (27.1%) met ABPA diagnostic criteria, consistent with a CPA/ABPA overlap phenotype. At the time of CPA diagnosis, 31 out of 45 individuals (69%) who fulfilled ABPA criteria already met these criteria. In the remaining cases, it was not possible to determine whether ABPA criteria were present earlier or developed during follow-up, as immunological assessments were performed as part of routine clinical care rather than a predefined longitudinal protocol. Among individuals with CPA who did not meet ABPA diagnostic criteria, 20 demonstrated *Aspergillus* sensitisation at the time of CPA diagnosis, while an additional 15 developed sensitisation during follow-up.

The mean age at CPA diagnosis was 53.1±13.7 years (range 20–85). The most frequent underlying pulmonary comorbidities were bronchiectasis (57.2%), sarcoidosis (27.7%) and asthma (21.1%). Median baseline body mass index (BMI) was 22.2 kg·m^−2^ (IQR 19.0–24.5). Full baseline characteristics are presented in table 1. Subsequent analyses compared CPA alone (n=121) and CPA/ABPA overlap (n=45).

**TABLE 1:** Selected demographic, clinical, radiological, microbiological, immunological and pulmonary function characteristics of the chronic pulmonary aspergillosis (CPA) cohort (n=166) stratified by allergic bronchopulmonary aspergillosis (ABPA) status

Variable	Total cohort	CPA	CPA/ABPA	OR (95% CI)	p-value
**Individuals, n**	166	121	45		
**Demographics**					
Age, years	60.8±13.0 (27–90)	59.8±12.8 (27–90)	63.6±13.1 (29–84)	1.024 (0.996–1.053)	0.10
Age at CPA diagnosis, years	53.1±13.7 (20–85)	52.3±13.7 (20–85)	55.1±13.5 (23–78)	1.015 (0.990–1.042)	0.24
Male sex	83 (50%)	63 (52.1%)	20 (44.4%)	0.737 (0.370–1.465)	0.38
**Symptoms**					
Sputum production	114 (68.7%)	81 (66.9%)	33 (73.3%)	1.358 (0.634–2.908)	0.43
Dyspnoea	105 (63.3%)	78 (64.5%)	27 (60%)	0.827 (0.409–1.670)	0.60
Cough	104 (62.7%)	80 (48.1%)	24 (53.3%)	0.586 (0.292–1.175)	0.13
Haemoptysis	97 (58.4%)	71 (58.7%)	26 (57.8%)	0.964 (0.482–1.928)	0.92
Weight loss	58 (34.9%)	44 (36.4%)	14 (31.1%)	0.790 (0.380–1.643)	0.53
Night sweats	29 (17.5%)	24 (19.8%)	5 (11.1%)	0.505 (0.180–1.417)	0.20
Fatigue	27 (16.3%)	14 (11.6%)	13 (28.9%)	3.105 (1.324–7.279)	**0.01**
Chest pain	26 (15.7%)	21 (17.4%)	5 (11.1%)	0.595 (0.210–1.687)	0.33
Fever	17 (10.2%)	16 (13.2%)	1 (2.2%)	0.149 (0.019–1.159)	0.07
Appetite loss	14 (8.4%)	10 (8.3%)	4 (8.9%)	1.063 (0.316–3.580)	0.92
**Radiological findings**					
Aspergilloma presence	147 (88.6%)	107 (88.4%)	40 (88.9%)	1.047 (0.354–3.094)	0.93
Bilateral involvement	64 (38.6%)	42 (34.7%)	22 (48.9%)	1.799 (0.899–3.602)	0.10
**Microbiology**					
*Aspergillus* spp. positive culture	68 (41.0%)	45 (37.2%)	23 (51.1%)	1.766 (0.885–3.524)	0.11
Azole resistance	13 (7.8%)	8 (6.6%)	5 (11.1%)	1.766 (0.546–5.712)	0.34
Pan-azole resistance	5 (3.0%)	1 (0.8%)	4 (8.9%)	11.707 (1.272–107.772)	**0.03**
**Comorbidities**					
Bronchiectasis	95 (57.2%)	68 (56.2%)	27 (60%)	1.169 (0.583–2.345)	0.66
** **Sarcoidosis	46 (27.7%)	42 (34.7%)	4 (8.9%)	0.184 (0.062–0.547)	**0.002**
** **Asthma	35 (21.1%)	11 (9.1%)	24 (53.3%)	11.429 (4.871–26.812)	**<0.001**
COPD	33 (19.9%)	21 (17.4%)	12 (26.7%)	1.732 (0.769–3.897)	0.19
Previous pulmonary TB	32 (19.3%)	25 (20.7%)	7 (15.6%)	0.707 (0.282–1.772)	0.46
Previous pneumothorax	28 (16.9%)	18 (14.9%)	10 (22.2%)	1.635 (0.690–3.875)	0.26
Pulmonary hypertension	26 (15.7%)	21 (17.4%)	5 (11.1%)	0.595 (0.210–1.687)	0.33
NTM-PD	24 (14.5%)	13 (10.7%)	11 (24.4%)	2.688 (1.103–6.549)	**0.03**
Interstitial lung disease	18 (10.8%)	17 (14.1%)	1 (2.2%)	0.139 (0.018–1.077)	0.06
Lung cancer	3 (1.8%)	2 (1.7%)	1 (2.2%)	1.352 (0.120–15.287)	0.81
Cystic fibrosis	2 (1.2%)	1 (0.8%)	1 (2.2%)	2.727 (0.167–44.547)	0.48
**Albumin and body mass index**					
Albumin at baseline g·L^−1^	40 (37–43)	40 (37–43)	40.2±3.9	1.031 (0.946–1.123)	0.49
Albumin at end-point g·L^−1^	41 (37–43)	41 (37–43)	40.2±4.4	1.019 (0.952–1.090)	0.60
BMI at baseline kg·m^−2^	22.2 (19.0–24.5)	22.3 (19–24.7)	20.5 (19.1–23.5)	0.975 (0.857–1.109)	0.70
BMI at end-point kg·m^−2^	20.6 (17.6–24.2)	21.3 (17.5–24.8)	20.6±3.2	0.952 (0.862–1.050)	0.33
**Smoking status**					
Smoking history	60 (67.4%)	47 (71.2%)	13 (56.5%)	0.526 (0.197–1.403)	0.20
Nonsmokers	29 (32.6%)	19 (28.8%)	10 (47.5%)	1.903 (0.713–5.078)	0.20
**Immunological markers**					
Total IgE at baseline kU·L^−1^	96 (32–515)	45 (15–145)	734 (302.3–2393)	1.001 (1.000–1.002)	**0.01**
≥1000 kU·L^−1^	16 (17.2%)	1 (1.6%)	15 (46.9%)	52.941 (6.518–430.019)	**<0.001**
Total IgE at end-point kU·L^−1^	67 (22–400)	39 (20–112.3)	578 (182–2100)	1.001 (1.000–1.002)	**0.004**
** **≥1000 kU·L^−1^	17 (14.8%)	1 (1.3%)	16 (41.0%)	52.174 (6.560–414.957)	**<0.001**
Total IgE variance from baseline kU·L^−1^	0 (−50.3–40.8)	0 (−12–22.8)	−30.5 (−1128.5–96.5)	0.999 (0.998–1)	0.06
*AF*-specific IgE at baseline kU·L^−1^	0.5 (0.1–7.2)	0.1 (0.01–0.6)	11.9 (5.1–28.3)	1.831 (1.385–2.421)	**<0.001**
*AF*-specific IgE at end-point kU·L^−1^	0.4 (0.03–3.9)	0.1 (0.02–0.9)	11.3 (3.8–31.6)	1.920 (1.419–2.598)	**<0.001**
*AF*-specific IgE variance from baseline kU·L^−1^	0 (−0.3–0.3)	0 (−0.1–0.1)	−0.3 (−6.1–15.4)	1.028 (0.989–1.069)	0.16
*AF*-specific IgG at baseline mgA·L^−1^	102 (58.7–165)	101 (54.6–148.5)	107 (63.2–169.8)	1.002 (0.996–1.008)	0.49
*AF*-specific IgG at end-point mgA·L^−1^	74.2 (44.4–128)	69.3 (38.1–134.5)	83.7 (59.0–122)	1.002 (0.996–1.007)	0.56
*AF*-specific IgG variance from baseline mgA·L^−1^	−14.9 (−51.4–4.2)	−16 (−52.1–6.8)	−9.8 (49.3–3.7)	0.999 (0.993–1.005)	0.66
**Lung function tests (% predicted)**					
FEV_1_ at baseline	58.7±22.7	60.1±22.9	50.7±20.8	0.980 (0.942–1.019)	0.31
FEV_1_ at end-point	60.7±23.0	60.3±23.1	62±23.9	1.003 (0.975–1.033)	0.83
FVC at baseline	71 (60.1–88.3)	71 (59.0–88.5)	75.7±16.0	1.003 (0.963–1.043)	0.90
FVC at end-point	80.4±28.0	80.2 (51.0–102.0)	89.4±21.8	1.015 (0.991–1.039)	0.24
*T*_LCO_ at baseline	44.2 (32.6–58.9)	44.2 (33.0–59.5)	42.5±15.1	0.979 (0.926–1.034)	0.44
*T*_LCO_ at end-point	51.6±23.2	50.6±23.1	55.7±24.2	1.010 (0.980–1.040)	0.53
**CPA interventions**					
Surgical excision	27 (16.3%)	20 (16.5%)	7 (15.6%)	0.930 (0.364–2.377)	0.88
Arterial embolisation	20 (12.1%)	15 (12.4%)	5 (11.1%)	0.883 (0.301–2.589)	0.82
**Antifungal treatment**					
Itraconazole	121 (72.9%)	85 (70.3%)	36 (80%)	1.694 (0.740–3.877)	0.21
Posaconazole	65 (39.2%)	43 (35.5%)	22 (48.9%)	1.735 (0.868–3.470)	0.12
Voriconazole	61 (36.8%)	40 (33.1%)	21 (46.7%)	1.772 (0.882–3.559)	0.11
Isavuconazole	28 (16.9%)	17 (14.1%)	11 (24.4%)	1.979 (0.845–4.638)	0.12
Casponfungin	75 (45.2%)	57 (47.1%)	18 (40%)	0.749 (0.374–1.500)	0.41
Micafungin	1 (0.6%)	0 (0%)	1 (2.2%)		>0.999
Amphotericin	12 (7.2%)	7 (5.8%)	5 (11.1%)	2.036 (0.611–6.778)	0.25

Data are presented as mean±sd, median (interquartile range) or n (%), as appropriate. Odds ratios (OR) and 95% confidence intervals (CI) were obtained from univariate binary logistic regression, with CPA as the reference group. For continuous variables, ORs represent the change in odds per-unit increase in the variable. A p-value <0.05 was considered statistically significant (in bold type). Percentages were calculated based on available data for each variable. The number of patients analysed for each variable were: demographics, symptoms, radiological findings, microbiology, comorbidities (n=166); albumin at baseline (n=130) and end-point (n=137); BMI baseline (n=72) and end-point (n=85); smoking status (n=89); total IgE baseline (n=93) and end-point (n=115); total IgE variance from baseline (n=69); *AF*-specific IgE baseline (n=107) and end-point (n=125); *AF*-specific IgE variance from baseline (n=88); *AF*-specific IgG baseline (n=143) and end-point (n=137); *AF*-specific IgG variance from baseline (n=72); FEV_1_ baseline (n=49) and end-point (n=51); FVC at baseline (n=47) and end-point (n=53); *T*_LCO_ at baseline (n=48) and end-point (n=51); CPA interventions (n=166); antifungal treatment (n=166). TB: tuberculosis; NTM-PD: nontuberculous mycobacterial pulmonary disease; BMI: body mass index; IgE: immunoglobulin E; *AF*: *Aspergillus fumigatus*; IgG: immunoglobulin G; FEV₁: forced expiratory volume in 1 s; FVC: forced vital capacity; *T*_LCO_: transfer factor of the lung for carbon monoxide.

#### Characteristics according to ABPA status

Asthma (53.3% *versus* 9.1%; p<0.001) and NTM-PD (24.4% *versus* 10.7%; p=0.03) were more frequent in the CPA/ABPA overlap group, while sarcoidosis was more common in CPA alone (8.9% *versus* 34.7%; p=0.002). Pan-azole resistance was higher in overlap (8.9% *versus* 0.8%; p=0.03). Fatigue was also more frequent (28.9% *versus* 11.6%; p=0.01), whereas other symptoms were similar. There was a trend towards older age (63.6±13.1 *versus* 59.8±12.8 years; p=0.10) and bilateral CPA (48.9% *versus* 34.7%; p=0.10) in overlap cases. Immunological markers differed significantly, with higher median total IgE and *A. fumigatus*-specific IgE levels at baseline and throughout follow-up in overlap individuals as expected. Median *A. fumigatus*-specific IgG levels were similar between groups (107 (IQR 63.2–169.8) *versus* 101 (IQR 54.6–148.5) mgA·L^−1^; p=0.49). No significant differences were seen in BMI, serum albumin, lung function or treatment interventions (surgery, embolisation, antifungal use).

Antifungal exposure was assessed longitudinally, including both initial therapy and agents used during follow-up. The most used antifungal agents included itraconazole, voriconazole and posaconazole. However, individuals with CPA/ABPA overlap more frequently received a higher number of antifungal agents (≥5 agents: 13.3% *versus* 4.1%) and were more likely to be exposed to more than two antifungal classes (9.1% *versus* 2.8%).

#### Multivariate analysis

In logistic regression using CPA alone as the reference, asthma (OR 10.163; 95% CI 3.880–26.575; p<0.001) and pan-azole resistance (OR 19.371; 95% CI 1.241–302.907; p=0.04) were independent predictors of ABPA overlap, whereas sarcoidosis was inversely associated (OR 0.223; 95% CI 0.064–0.7712; p=0.02) (table 2). Increasing age was not significantly associated with ABPA overlap (OR 1.030 per year; 95% CI 0.996–1.065; p=0.08).

**TABLE 2:** Multivariate logistic regression analysis of factors associated with allergic bronchopulmonary aspergillosis (ABPA) overlap among chronic pulmonary aspergillosis (CPA) individuals

Variable	OR (95% CI)	p-value
**Asthma**	10.163 (3.880–26.575)	**<0.001**
**Sarcoidosis**	0.223 (0.064–0.771)	**0.02**
**Pan-azole resistance**	19.371 (1.241–302.907)	**0.04**
**Age, years**	1.030 (0.996–1.065)	0.08
**Interstitial lung disease**	0.127 (0.011–1.520)	0.10
**Bilateral involvement**	0.900 (0.350–2.318)	0.83
**NTM-PD**	0.954 (0.305–2.980)	0.94

CPA without ABPA was used as the reference group. Variables with p<0.10 in univariate analysis were included in multivariate logistic regression analysis. Odds ratios (OR) with 95% confidence intervals (CI) are presented. Statistical significance was defined as p-value <0.05 (in bold type). NTM-PD: nontuberculous mycobacterial pulmonary disease.

### Survival analysis

**FIGURE 1:**
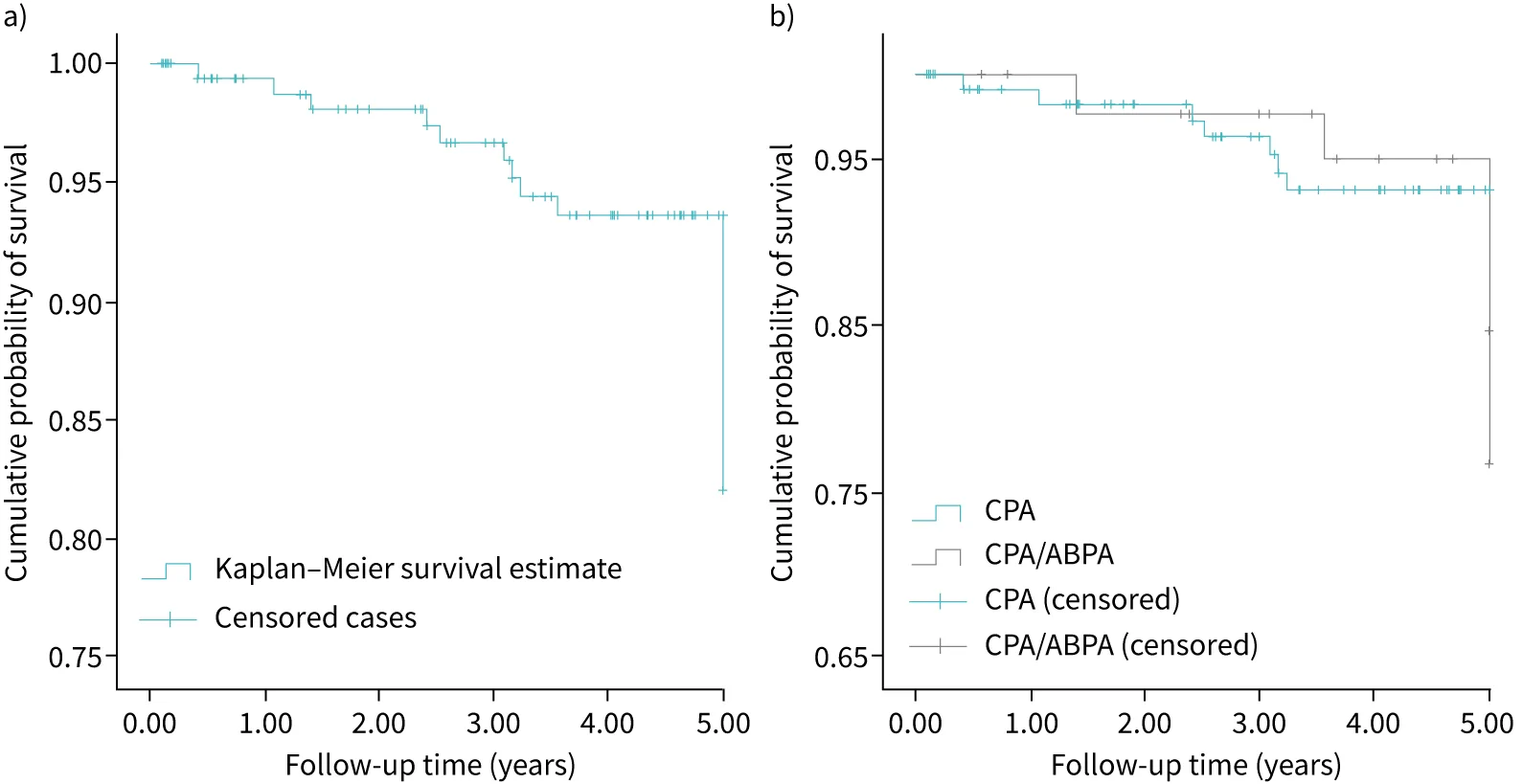
Kaplan–Meier survival curves over a 5-year follow-up period. a) Overall chronic pulmonary aspergillosis (CPA) cohort (n=166). The estimated 5-year survival probability was 82% (95% CI: 74.94–89.06). Marks represent censored observations, with censoring at the date of last contact or end of the observation window. Most deaths occurred after the third year of follow-up. b) Survival stratified by allergic bronchopulmonary aspergillosis (ABPA) status. The 5-year survival probability was 84.6% (95% CI: 76.76–92.44) in the CPA only group (n=121) and 76.6% (95% CI: 62.29–90.91) in the CPA/ABPA group (n=45). No statistically significant difference was observed between groups (log-rank test, p=0.44). Crosses indicate censored observations.

Overall 5-year survival probability was 82% (95% CI 74.94–89.06; figure 1a). Median follow-up was 5.0 years (IQR 3.22–5.00), with mean survival of 4.83 years (95% CI 4.72–4.95). Median time from diagnosis to death was 5.5 years (IQR 2.75–9.67). 1- and 3-year survival were 98.7% (95% CI 96.94–100.00) and 95.9% (95% CI 92.76–99.04). No significant survival difference was observed between CPA/ABPA overlap and CPA alone (76.6% (95% CI: 62.29–90.91) *versus* 84.6% (95% CI 76.80–92.40); p=0.44; figure 1b). During follow-up, 13 deaths (10.7%) occurred in the CPA-only group and 8 (17.8%) in the CPA/ABPA group. Median time to death was 3.24 years (IQR 2.52–5.00) in CPA alone and 5.00 years (IQR 4.28–5.00) in overlap cases.

### Predictors of mortality

In univariable Cox regression, older age at CPA diagnosis predicted higher mortality (HR 1.044 per year, 95% CI 1.010–1.079; p=0.01). Lung cancer conferred the highest risk (HR 20.665, 95% CI 2.290–186.488; p=0.01). Lower BMI at end-point (HR 0.826 per kg·m^−2^, 95% CI 0.701–0.973; p=0.02) and lower serum albumin (baseline HR 0.881 per g·L^−1^, 95% CI 0.795–0.976; p=0.004; end-point HR 0.941 per g·L^−1^, 95% CI 0.903–0.980; p=0.004) were associated with increased mortality.

Although, CPA/ABPA overlap was not associated with higher death risk (HR 1.396, 95% CI 0.578–3.369; p=0.46), elevated Th2 immunological markers correlated with mortality: total IgE ≥1000 kU·L^−1^ at diagnosis (HR 3.836, 95% CI 1.110–13.250; p=0.03), elevated *A. fumigatus*-specific IgE at end-point (HR 1.012, 95% CI 1.001–1.023; p=0.023) and longitudinal increase in *A. fumigatus*-specific IgE (HR 1.020 per unit variance, 95% CI 1.003–1.037; p=0.02). Baseline *A. fumigatus*-specific IgG also predicted mortality (HR 1.012 per mgA·L^−1^, 95% CI 1.004–1.020; p=0.01). Full univariate regression results are provided in supplementary table S1.

Analysing all individuals with CPA through multivariable Cox regression (table 3), serum albumin at end-point, however, was the only independent mortality predictor (HR 0.724, 95% CI 0.603–0.869; p=0.001), while age, sex and immunological markers were not significant. AF-specific IgE at end-point was not significantly associated with mortality (HR 1.012, 95% CI 0.999–1.025; p=0.07).

**TABLE 3:** Multivariable Cox proportional hazards regression analysis of predictors of mortality in chronic pulmonary aspergillosis (CPA) individuals

Variable	HR (95% CI)	p-value
**Age at diagnosis, years**	0.984 (0.924–1.048)	0.61
**Albumin at end-point, g·L^−1^**	0.724 (0.603–0.869)	**0.001**
**Male sex**	3.585 (0.738–17.440)	0.11
***AF*-specific IgG at baseline**	1.005 (0.993–1.016)	0.41
**Total IgE at end-point ≥1000 kUA·L^−1^**	2.391 (0.589–9.704)	0.22
***AF*-specific IgE at end-point**	1.012 (0.999–1.025)	0.07

Variables included were those with p<0.10 in univariable analysis, after exclusion of collinear and low-prevalence factors. Hazards ratios (HR) and 95% confidence intervals (CI) are shown. HRs are adjusted for age at diagnosis (per 1-year increase), sex (reference group: female), serum albumin at end-point (g·L^−1^), *Aspergillus fumigatus*-specific IgG at baseline (mgA·L^−1^), *A. fumigatus*-specific IgE at end-point (kUA·L^−1^) and total IgE at end-point ≥1000 kUA·L^−1^ (reference group: IgE <1000). A p-value <0.05 was considered statistically significant (in bold type). *AF*: *Aspergillus fumigatus*; IgG: immunoglobulin G; IgE: immunoglobulin E.

## Discussion

In this large retrospective cohort study, we show that nearly one-third of CPA patients fulfilled diagnostic criteria for ABPA with CPA/ABPA overlap demonstrating a distinct clinical phenotype associated with specific comorbidities, increased incidence of azole resistance and with *Aspergillus* sensitisation linked to increased mortality risk. These findings suggest that ABPA and/or *Aspergillus* sensitisation complicating CPA is more frequent than previously recognised and underlines the importance of better understanding of the underlying pathophysiology in CPA and the relevance of T2 inflammation.

Although ABPA and CPA have traditionally been regarded as distinct entities, these findings suggest an immunological interplay with potential impact on disease progression. In our cohort, we reported a higher proportion with ABPA/CPA overlap than previously reported which may in part reflect adoption of the 2024 revised ISHAM ABPA criteria, which lowers the total IgE threshold and includes individuals with nonasthmatic or cystic fibrosis predisposing conditions [1, 18–20]. Although asthma was present in just over half of individuals with CPA/ABPA overlap, current ISHAM criteria recognise that ABPA may occur in a broader range of underlying lung diseases, including bronchiectasis, COPD and CPA.

Asthma and NTM-PD were more common in ABPA/CPA overlap, whereas sarcoidosis was less frequent perhaps reflecting the strong link between ABPA and type-2–driven airway disease, alongside the potential immunological anergy previously noted in sarcoidosis [10, 12, 19, 21, 22]. Although there was marked individual variability in clinical symptoms, a higher frequency of fatigue was also noted in individuals with ABPA/CPA overlap. Previous studies have shown that fatigue is a major component of impaired health status in individuals with CPA. Whilst associated with heightened immune activation in other settings, further studies are required to better understand immunological drivers of symptoms in CPA.

Our study also identified a higher frequency of azole resistance among individuals with CPA/ABPA overlap. Although antifungal exposure was assessed longitudinally, including both initial and subsequent therapies, detailed data on treatment duration and cumulative exposure were not available, limiting interpretation of this finding. Importantly, there were no clear differences between CPA and CPA/ABPA groups in terms of the classes of antifungal agents used. However, a subset of individuals with CPA/ABPA overlap was exposed to a higher number of antifungal agents and to multiple antifungal classes over time, suggesting greater treatment complexity in this subgroup. Azole resistance in CPA is likely multifactorial, influenced not only by antifungal exposure but also by environmental factors, fungal burden and host–pathogen interactions, alongside suboptimal serum drug concentrations in the presence of cavitary aspergillomas [10, 23, 24]. Whether a T2 primed airway micro-environment may represent a favourable environment for the development or selection of resistant *A. fumigatus* strains is not clear and raises important questions about host–pathogen interactions requiring further investigation. As often noted, *Aspergillus* spp. was cultured in ∼40% of CPA individuals reflecting the limited sensitivity of fungal culture and likely underestimating the scale and/or impact of azole resistance in clinical practice.

In our cohort, the estimated 5-year survival probability was 82%, which aligns with recent literature reporting long-term mortality rates of 27–32% in CPA patients [3]. When stratified by ABPA status, there was no significant difference in 5-year survival with CPA/ABPA overlap compared with CPA alone. In univariable analysis, however, alongside established factors associated with poorer survival such as older age at diagnosis, poor nutritional status and the presence of lung cancer, a raised total IgE ≥1000 kU·L^−1^ at diagnosis, elevated *A. fumigatus*-specific IgE at study end-point and variance over the study duration were also associated with increased mortality. This suggests that T2 inflammation may be associated with progressive pathophysiology and worse outcome. Whether this simply reflects increased bioburden and antigen exposure and an indicator of poor prognosis or is directly impacting on disease course is as yet unknown and requires further study.

Notably, total IgE ≥1000 kU·L^−1^ at diagnosis also predicted higher mortality even after adjusting for ABPA status, indicating perhaps a reflection of broader immune dysregulation or systemic inflammation beyond classical ABPA. In our multivariable model, serum albumin at end-point remained an independent predictor of mortality after adjustment for relevant clinical and immunological variables, reinforcing its role as a robust prognostic marker in CPA (HR 0.72, p=0.001), as demonstrated in other respiratory illnesses such as COPD and pulmonary fibrosis [4, 25]. However, albumin is a nonspecific marker influenced by multiple factors, including nutritional status, comorbidities and systemic inflammation. Although measurements in our cohort were obtained during routine outpatient follow-up, the observed association with mortality should be interpreted within this broader clinical context.

Taken together, these results suggest individuals with CPA/ABPA overlap represent a clinically and immunologically distinct subgroup with differing pathophysiology and potential impact on disease progression. The presence and evolution of *Aspergillus* sensitisation in individuals without ABPA supports the concept of a spectrum of host–fungal interaction in CPA. Notably, ABPA can progress to CPA, particularly in individuals with long-standing disease and structural lung damage [15]. More recently, several authors have proposed that ABPA and CPA may represent points along a disease continuum. ∼14% of CPA cases are estimated to arise from prior ABPA, and the presence of fungal balls in ABPA patients has been associated with a worse prognosis [1, 16, 26]. Epidemiological data reinforce this association, with global estimates indicating that up to 20% of ABPA patients may go on to develop CPA, while UK data report that 12–14% of CPA cases occur in the context of ABPA [19, 27]. Although this transition is recognised, the temporal sequence and underlying mechanisms remain poorly defined and remain difficult to establish, particularly given the delayed diagnosis of CPA [1, 15]. In our cohort, a substantial proportion of individuals already fulfilled ABPA criteria at the time of CPA diagnosis, while in others it was not possible to determine whether these criteria developed earlier or during follow-up. In addition, *Aspergillus* sensitisation was observed both at diagnosis and emerging over time in individuals without ABPA, supporting the concept of a dynamic host–fungal interaction. A clearer understanding of this complication could help clinicians identify ABPA patients at risk of progression, support earlier escalation of antifungal therapy, and ultimately reduce morbidity and mortality [15].

Previous cytokine profiling has shown that ABPA is characterised by a Th2-skewed response with elevated interleukin (IL)-5 and IL-13, whereas CPA exhibits higher levels of proinflammatory cytokines such as IL-33 and tumour necrosis factor, together with lower IL-5 [16]. IL-10 and sCD40L have been reported to be elevated in both ABPA and CPA [28]. Importantly, in cases where CPA arises from ABPA, it has been hypothesised that chronic allergic inflammation may facilitate fungal persistence and progression to tissue destruction. In this context, the IL-10/IL-5 ratio has been proposed as a supplemental biomarker, with good specificity for CPA [16]. Furthermore, CPA development seems to require both impaired antifungal defence and defective control of inflammation [29].

This is pertinent when considering direct therapeutic implications, particularly regarding the use of corticosteroid therapy which is a cornerstone of ABPA management [16]. Whether prolonged use impairs antifungal defences and favours transformation to progressive cavitary infection is unclear. Recent real-world studies have highlighted the ability of novel T2 targeted monoclonal antibody therapy to improve outcome in ABPA and minimise corticosteroid use [30]. Whether this is likely to reduce CPA progression in ABPA is not clear. Equally, targeted T2 therapeutics may present a plausible strategy in CPA to revert deleterious effects of T2 inflammation on epithelial barrier integrity and production of antimicrobial peptides and improve host immune antifungal response [31–33]. The role of T2 targeted monoclonal antibodies in CPA is however again not clear and requires further research.

Although well described, large well-characterised CPA cohorts remain relatively scarce, particularly those addressing the overlap with ABPA. Despite the size of the cohort in our study, as a retrospective single centre cohort study, there are limitations that should be acknowledged. The study design introduces the potential for selection bias and is inherently associated with incomplete or missing data. The number of deaths and size of cohort also limited the power of multivariable analyses, constraining the ability to fully explore independent predictors of mortality. Moreover, mortality in CPA is often multifactorial, reflecting advanced respiratory disease and underlying comorbidities rather than a single attributable cause. In this study, the retrospective design and incomplete availability of cause-of-death data limited the ability to systematically determine cause-specific mortality, and this should be considered when interpreting mortality outcomes. Furthermore, the temporal relationship between CPA and ABPA could not be definitively established due to the retrospective design and lack of systematic longitudinal assessment. In some cases, absence of documented ABPA criteria at diagnosis may reflect incomplete data capture rather than true absence. Data on corticosteroid exposure were not systematically collected and detailed data on antifungal treatment duration and cumulative exposure were not available. Detailed mechanistic immunological data were not available in this retrospective cohort. In particular, cytokine profiling and markers such as the IL-10/IL-5 ratio were not assessed, limiting our ability to determine whether CPA/ABPA overlap reflects a distinct immunopathological phenotype or to directly support differences in Th1/Th2 pathways. Nevertheless, our findings provide important insights into the role of *Aspergillus* sensitisation and ABPA overlap in CPA and highlight the need for prospective, multicentre studies with standardised follow-up and comprehensive outcome reporting.

To summarise, in this study, we provide further insight into the prognostic role of *Aspergillus* sensitisation and ABPA overlap in CPA, and detail that CPA/ABPA overlap represents a clinically distinct subgroup with higher rates of asthma, fungal sensitisation and antifungal resistance. Although ABPA overlap was not associated with increased 5-year mortality risk, *Aspergillus* sensitisation and T2 inflammation was associated with mortality, suggesting a potential role as a prognostic biomarker. The presence of overlap syndromes challenges the traditional dichotomy between ABPA and CPA, highlighting the need for phenotype-driven approaches and research to understand impact on disease pathophysiology alongside large multicentre prospective studies to determine whether tailored therapy can improve outcomes.

## Supplementary material

**Please note:** supplementary material is not edited by the Editorial Office, and is uploaded as it has been supplied by the author. Supplementary material 01540-2025.SUPPLEMENT
