# Supplementary material for: Impact of allergic bronchopulmonary aspergillosis overlap in chronic pulmonary aspergillosis
Source: ERJ Open Res. 2026 Sep 21;12(5):01540-2025. doi: 10.1183/23120541.01540-2025 (PMC13591302; doi:10.1183/23120541.01540-2025)
Supplement: Supplementary file 1 [file 01540-2025.SUPPLEMENT.pdf]

SUPPLEMENTARY MATERIAL

Variables	Survived (n=145)	Dead (n=21)	HR (95% CI)	p-value
**Demographics**				
Male sex, n (%)	69 (47.6%)	14 (66.7%)	2.427 (0.979-6.020)	0.06
Age, years	61 (51-69.5)	66.6 $\pm$ 12.6	1.037 (1.000-1.076)	0.05
**Age at CPA diagnosis, years**	52.3 $\pm$ 13.3	58.6 $\pm$ 15.0	**1.044 (1.010-1.079)**	**0.01**
**CPA/ABPA overlap, n (%)**	37 (25.5%)	8 (38.1%)	1.396 (0.578-3.369)	0.46
**Symptoms, n (%)**				
Sputum production	98 (67.6%)	16 (76.2%)	1.191 (0.436-3.252)	0.73
Cough	90 (62.1%)	14 (66.7%)	1.098 (0.443-2.721)	0.84
Dyspnoea	88 (60.7%)	17 (81.0%)	2.589 (0.871-7.695)	0.09
Haemoptysis	88 (60.7%)	9 (42.9%)	0.568 (0.240-1.349)	0.20
Weight loss	51 (35.2%)	7 (33.3%)	0.944 (0.381-2.339)	0.90
Night sweats	26 (17.9%)	3 (14.3%)	0.820 (0.242-2.786)	0.75
Fatigue	26 (17.9%)	1 (4.8%)	0.280 (0.038-2.087)	0.21
Chest pain	24 (16.6%)	2 (9.5%)	0.508 (0.118-2.182)	0.36
Fever	14 (9.7%)	3 (14.3%)	1.568 (0.462-5.323)	0.47
Appetite loss	11 (7.6%)	3 (14.3%)	1.861 (0.548-6.320)	0.32
**Radiological findings**				
Aspergilloma presence	126 (86.9%)	21 (100%)	23.900 (0.056-10220.050)	0.31
Bilateral involvement	52 (35.9%)	12 (57.1%)	1.749 (0.737-4.151)	0.21
**Microbiology**				
Aspergillus spp. positive culture	56 (38.6%)	12 (57.1%)	1.691 (0.712-4.014)	0.23
Azole resistance	12 (8.3%)	1 (4.8%)	0.426 (0.057-3.173)	0.41
Pan-azole resistance	5 (3.5%)	0 (0%)	0.046 (0.000-439.898)	0.51
**Comorbidities**				
Bronchiectasis	81 (55.9%)	14 (66.7%)	1.524 (0.615-3.778)	0.36
Sarcoidosis	40 (27.6%)	6 (28.6%)	0.933 (0.362-2.406)	0.89
Asthma	31 (21.4%)	4 (19.1%)	0.758 (0.255-2.255)	0.62
Previous pulmonary TB	29 (20%)	3 (14.3%)	0.876 (0.258-2.978)	0.83
COPD	28 (19.3%)	5 (23.8%)	1.903 (0.692-5.232)	0.21
Previous Pneumothorax	23 (15.9%)	5 (23.8%)	1.518 (0.556-4.143)	0.42
Pulmonary Hypertension	21 (14.5%)	5 (23.8%)	1.626 (0.596-4.439)	0.34
NTM-PD	21 (14.5%)	3 (14.3%)	0.843 (0.248-2.864)	0.78
Interstitial lung disease	17 (11.7%)	1 (4.8%)	0.615 (0.082-4.582)	0.64
**Lung cancer**	2 (1.4%)	1 (4.8%)	**20.665 (2.290-186.488)**	**0.01**
Cystic Fibrosis	2 (1.4%)	0 (0%)	0.048 (0.000-82960.942)	0.68

**Albumin and Body Mass Index**				
BMI at baseline (per 1-unit increase)	22.1 (19.0-24.8)	21.8 ± 3.8	0.944 (0.808-1.104)	0.47
**BMI at endpoint (per 1-unit increase)**	21.9 (17.7-24.7)	18.7 (16.8-20.4)	**0.826 (0.701-0.973)**	**0.02**
**Albumin at baseline, g/L**	41 (37-43)	40 (36-42)	**0.881 (0.795-0.976)**	**0.02**
**Albumin at endpoint, g/L**	41.7 (37.9-44)	35.8 ± 4.8	**0.941 (0.903-0.980)**	**0.004**
**Smoking Status**				
Smoking history	52 (66.67%)	8 (72.73%)	1.320 (0.350-4.975)	0.68
Non-smokers	26 (33.33%)	3 (27.27%)	0.758 (0.201-2.857)	0.68
**Immunological Markers**				
Total IgE at diagnosis, kU/L	151 (36.8-437)	11.3 ± 1021.3	1.000 (1.000-1.000)	0.28
**Total IgE at diagnosis ≥ 1000 kU/L**	15 (18.3%)	5 (50%)	**3.836 (1.110-13.250)**	**0.03**
Total IgE at baseline, kU/L	90 (32-345.5)	470.5 (22.5-1932.3)	1.000 (1.000-1.000)	0.25
Total IgE at baseline ≥ 1000 kU/L	12 (14.8%)	4 (33.3%)	2.748 (0.827-9.126)	0.10
Total IgE at endpoint, kU/L	64 (20-319)	312 (49-1665.8)	1.000 (1.000-1.000)	0.80
Total IgE at endpoint ≥ 1000 kU/L	13 (12.9%)	4 (28.6%)	2.977 (0.933-9.501)	0.07
Total IgE variance from diagnosis, kU/L	-12 (-340.5-18.5)	-326.5 ± 681.6	1.000 (0.999-1.001)	0.74
Total IgE variance from baseline, kU/L	0 (-48-29.5)	4.5 (-279-337.3)	1.000 (0.999-1.000)	0.78
AF-specific IgE at baseline, kU/L	0.40 (0.04-5.3)	3.3 (0.2-19.2)	1.014 (0.989-1.040)	0.27
***AF*-specific IgE at endpoint, kU/L**	0.41 (0.03-3.6)	2.4 (0.1-31.8)	**1.012 (1.001-1.023)**	**0.03**
***AF*-specific IgE variance from baseline, kU/L**	0 (-0.4-0-30)	0.9 (-0.1-17.6)	**1.020 (1.003-1.037)**	**0.02**
**AF-specific IgG at baseline, kU/L**	97 (57.9-145.5)	148 (82-193)	**1.012 (1.004-1.020)**	**0.01**
AF-specific IgG at endpoint, kU/L	69.55 (45.9-126)	97.0 ± 73.7	1.003 (0.996-1.011)	0.39
AF-specific IgG variance from baseline, kU/L	-19 (-43.7-13)	-42.5 ± 57.7	0.997 (0.989-1.006)	0.51
**Lung Function Tests (% predicted)**				
FEV_1 at baseline	60.1 ± 22.9	51.7 ± 21.6	0.990 (0.955-1.025)	0.56
FEV_1 at endpoint	61.8 ± 23.0	53.5 ± 23.4	0.990 (0.954-1.027)	0.58
FVC at baseline	71 (59-91)	71.6 ± 10.3	0.996 (0.958-1.035)	0.83
FVC at endpoint	82 ± 28.8	69.8 ± 20.4	0.988 (0.959-1.018)	0.44
TLCO at baseline	49.5 ± 18.6	36.5 ± 7.4	0.946 (0.886-1.011)	0.10
TLCO at endpoint	54.2 ± 22.6	31.2 (23.7-32.4)	0.960 (0.917-1.004)	0.08
**CPA interventions**				
Surgical excision	25 (17.2%)	2 (9.5%)	0.548 (0.128-2.351)	0.42
Arterial embolization	19 (13.1%)	1 (4.8%)	0.439 (0.059-3.275)	0.42
**Antifungal treatment, n (%)**				
Itraconazole	105 (72.4%)	16 (76.2%)	0.991 (0.363-2.705)	0.99
Casponfungin	65 (44.8%)	10 (47.6%)	0.972 (0.412-2.288)	0.95
Posaconazole	54 (37.2%)	11 (52.4%)	1.585 (0.673-3.731)	0.29
Voriconazole	53 (36.6%)	8 (38.1%)	0.845 (0.349-2.044)	0.71
Isavuconazole	24 (16.6%)	4 (19.1%)	1.029 (0.346-3.059)	0.96
Amphotericin	11 (7.6%)	1 (4.8%)	0.550 (0.074-4.100)	0.56
Micafungin	1 (0.7%)	0 (0%)	0.049 (0.000-30350527.4)	0.77

Supplementary Table 1. Univariate Cox proportional hazards regression analysis of predictors of mortality in CPA individuals.
Hazard ratios (HR), 95% confidence intervals (CI), and p-values are shown for selected variables. Continuous variables were analysed per unit increase; categorical variables are shown as binary indicators. Significant results ($p < 0.05$) are shown in bold.
